# Client-based evaluation of the effects of localized vibration therapy on pain and mobility scores in dogs with radiographic bilateral hip dysplasia

**DOI:** 10.3389/fvets.2024.1424373

**Published:** 2024-08-21

**Authors:** Kristal F. Turner, Sherman O. Canapp, Debra A. Canapp, Angela M. Sutton, Allyson Canapp, Isabel A. Jimenez, Joyce Gerardi

**Affiliations:** ^1^Canapp Sports Medicine, Oakland, MD, United States; ^2^mOcean mobility + wellness for animals, Jacksonville Beach, FL, United States; ^3^Johns Hopkins University School of Medicine, Baltimore, MD, United States; ^4^Synergy Integrative Speciality Veterinary Clinic, New Bern, NC, United States

**Keywords:** vibration, vibration therapy, localized vibration therapy, pain management, hip dysplasia, vibration treatment, canine (dog), rehabilitation

## Abstract

**Introduction:**

This study evaluated the effects of localized vibration (LV) in 37 dogs with bilateral hip dysplasia (HD). HD is a common cause of lameness in dogs, and is a contributory factor to osteoarthritis, which can reduce the dog’s overall quality of life.

**Materials and methods:**

This was a multi-center, prospective survey-based study of 37 dogs with bilateral HD and no prior history of surgical management. Dogs were given LV therapy daily for 14 consecutive days using the same commercially available handheld vibration device. Canine Brief Pain Index (CBPI) data was collected prior to the initiation of therapy, then for 14 days following daily LV therapy. The dogs’ medications, supplements, additional rehabilitation modalities, and activity level were unchanged during the study period. Baseline CBPI pain severity and pain interference scores were compared to scores after 7 or 14 days of LV.

**Results:**

There were significant decreases in average pain severity and average pain interference CBPI scores in response to 7 and 14 days of therapy compared to baseline. When response to therapy was defined as a decrease in both pain severity score and pain interference score, 62% (23/37) of dogs responded to therapy at 7 days of treatment and 73% (27/37) responded at 14 days of treatment. Of the individuals that responded to treatment at 7 days, 91% (21/23) continued to respond at 14 days.

**Conclusion:**

Daily LV resulted in a significant reduction in CBPI scores in 73% of dogs with bilateral HD in this study. Randomized and blinded studies should be performed to further assess daily LV as a treatment modality for canine HD.

## Introduction

Canine hip dysplasia (HD) is a common cause of lameness in dogs of various sizes and breeds (1). This condition has a multifactorial etiology with genetic, epigenetic, and environmental components (2). The pathogenesis of HD involves initial joint laxity that leads to repeated subluxation of the femoral head, which precipitates joint inflammation, fraying of associated ligaments, erosion of articular cartilage, and structural changes to the hip joint. This cycle leads to gait abnormalities, osteoarthritis, pain, and further structural changes (3), which, in turn, can cause a reduction in the dog’s overall mobility and quality of life (4). Clinical signs visible to the dog’s owner include gait abnormalities/lameness, as well as reluctance in rising, jumping, running, or climbing stairs (5, 6).

Vibration therapy has been shown to benefit humans with a range of conditions, although most research in this area has focused primarily on whole body vibration (WBV). WBV typically consists of performing static or dynamic exercises on a vibrating platform (7). WBV has been shown to improve comfort and mobility in human patients with osteoarthritis (8–12). However, limitations of WBV include the difficulty of targeting specific muscles, the potential for attenuation of the vibratory signal by the time it reaches the intended muscle (13), and the cost and accessibility of specialized equipment. Localized vibration (LV) with a handheld vibration device mitigates some of these drawbacks and has demonstrated initial therapeutic benefits, including analgesic effects (14, 15), enhanced neuromuscular function (16–18), improved muscle strength (19–21) and increased flexibility (22) in humans, although research is more limited. LV therapy allows targeted application of vibration to specific muscle groups, and portable devices allow this therapy to be applied in home settings, thus increasing accessibility, compliance, frequency of use, and cost efficacy.

Research on the use of vibration therapy in dogs is limited. WBV does not appear to result in adverse effects on biochemical and physiologic parameters in healthy dogs (23–27). WBV has also been evaluated in dogs with clinical conditions and has been shown to enhance pulmonary gas exchange (28–30). A case report by Santos et al. (31) showed spontaneous opening of the cervix in a female dog with metritis after a single WBV session. However, WBV has only been assessed in the context of canine orthopedic disease in two studies. Gomes et al. (32) evaluated the effects of long-term WBV in dogs with bilateral HD, reporting improvements in hindlimb muscle mass as assessed by several objective measures, as well as reduction in pain as assessed by an owner-completed questionnaire. Martins et al. (33) demonstrated that dogs with HD-associated osteoarthritis had better clinical outcomes when they received one intra-articular injection of hyaluronic acid combined with WBV, compared to dogs who received hyaluronic acid alone. In this study, we hypothesized that the use of LV therapy in dogs with bilateral HD would result in decreased pain severity and pain interference as assessed using the Canine Brief Pain Inventory (CBPI) (34).

## Materials and methods

### Recruitment and enrollment

Thirty-seven dogs in the USA, Canada, and New Zealand were enrolled into the study through direct recruitment of owners via social media groups and through referral from rehabilitation specialists. Enrollment criteria for the study included: (1) a prior diagnosis of bilateral HD from a veterinarian; (2) recent bilateral hip radiographs; (3) no prior history of surgical management (e.g., total hip replacement or femoral head ostectomy). Signalment data (sex, age, breed) and weight was obtained for each dog. Enrolled dogs ranged in age from 1–13 years, with a mean of 7.1 years and a median of 7 years. Dogs ranged in weight from 9.2–72.7 kg, with a mean of 30.5 kg and a median of 28.2 kg. Sixteen different breeds were represented from five AKC breed groups, primarily from the Herding Group (e.g., Border Collies, Australian Shepherds, and German Shepherd Dogs), Sporting Group (e.g., Labrador Retriever, Golden Retriever) and Working Group (e.g., Bernese Mountain Dog, Great Dane, Doberman Pinscher). Overall, the majority of participants were medium, large, and giant breed dogs. Owners were instructed not to start or stop any medications, supplements, or additional rehabilitation modalities during the study period and to continue their dog’s normal activity level and exercise regimen throughout the study period. This study was performed with pre-approval from the Veterinary Orthopedic Sports Medicine Research Committee and with client consent.

### Radiograph scoring

One of the authors (SOC) evaluated hip radiographs for each dog in the study to confirm the diagnosis of HD and confirm enrollment criteria. To subjectively categorize dogs by severity of HD, SOC scored radiographs from 1–3 (1 = mild, 2 = moderate, 3 = severe) based on definitions derived from the OFA scoring system (35). Briefly, HD was classified as mild when significant coxofemoral subluxation was present, with a shallow acetabulum providing partial coverage of the femoral head, resulting in visibly increased joint space, and no arthritic changes present. HD was classified as moderate when the femoral head was barely seated into a shallow acetabulum, and secondary osteoarthritic changes were observed, with remodeling of the femoral neck and head, osteophytosis of the acetabular rim, and sclerosis. HD was classified as severe when the femoral head was partially or fully luxated out of a shallow acetabulum, and marked osteoarthritic changes were observed. A cumulative HD score was calculated for each dog by adding the HD scores for the left and right hip. This score was used as a subjective measure of HD severity to assess the potential influence of HD severity on response to therapy.

### Vibration therapy

**Figure 1:**
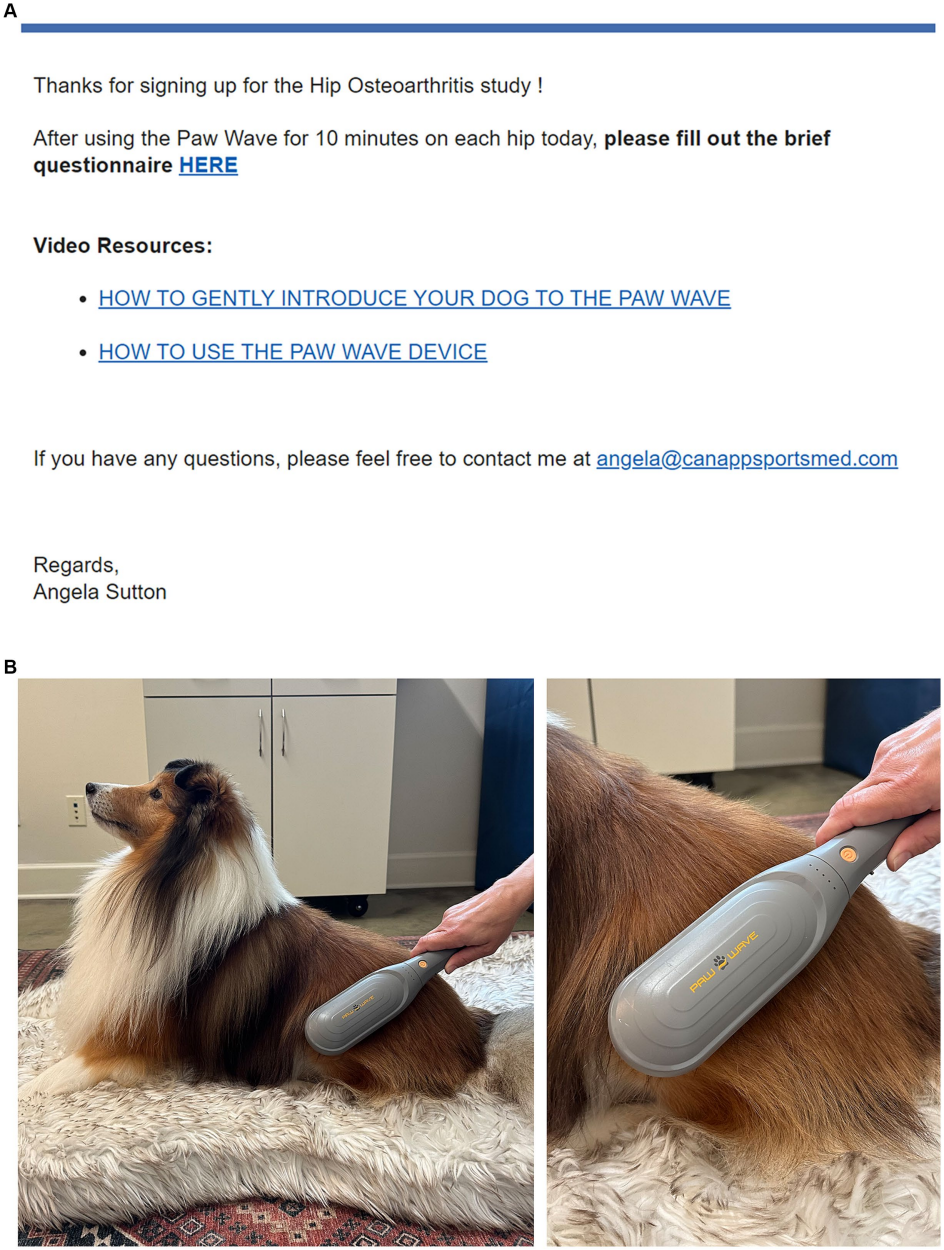
**(A)** Owners received daily reminder emails throughout the 14-day study period with the link to the online CBPI form and video resources on device use. **(B)** Owners were sent a commercially available handheld vibration device and were instructed to apply the device, in accordance with training videos, for 10 min per hip at the highest setting (120 Hz frequency, 1 mm amplitude), daily for 14 consecutive days.

Owners were sent a commercially available handheld vibration device (PawWave Buzz, Pado Inc., Valencia, CA 91355). Owners received written instructions and were required to watch a video resource on the use of the device prior to the start of the study. Owners received daily reminder emails during the study period (Figure 1A) and were instructed to apply the vibration device in accordance with the video for 10 min per hip (Figure 1B), at the highest setting (120 Hz frequency, 1 mm amplitude), daily for a total of 14 consecutive days during the study period.

### Canine Brief Pain Inventory

The CBPI was developed by Dr. Dorothy Cimino Brown at the University of Pennsylvania (34). The CBPI allows pet owners to rate the severity of their dog’s pain (4 questions) and the perceived interference of pain with normal functions (6 questions) on a scale of 1–10. The CBPI has been validated for the assessment of chronic pain, particularly osteoarthritis (34, 36). In the current study, owners were provided with an online form to record the CBPI. Owners were instructed to complete the CBPI prior to the start of the study to establish a baseline, and then once after each vibration therapy session for the study period of 14 days (37).

### Statistical analysis

Statistical analyses were run in Prism 10.2.3. Unless otherwise stated, the significance level for all statistical comparisons was alpha = 0.05. CBPI responses were used to calculate a pain severity score (average score of the four pain severity questions) and pain interference score (average score of the six pain interference questions) as previously described in Brown et al. (36). The change over time in pain severity and pain interference scores was assessed at days 0, 7, and 14 using repeated measures one-way ANOVA, with Dunnett’s multiple comparisons test to compare scores at day 7 and day 14 against baseline scores, and Geisser–Greenhouse correction. In addition, the same statistical methods were used to assess the effect of vibration therapy on scores from several individual questions from the CBPI, regarding the interference of pain in general activity, rising, walking, running, and climbing. Response to therapy was defined as individuals for which both pain severity score and pain interference score decreased between days 0 and 14. A paired two-tailed Mann–Whitney test was used to evaluate for differences in the severity of HD score between participants’ left and right hips.

To assess sex, age, weight, cumulative HD score, baseline pain severity score, and baseline pain interference score as potential predictors of response to therapy at 7 days or at 14 days, individual univariate logistic regression analyses were run to assess the relationship between the outcome and each individual predictor, using a significance level of alpha = 0.10 (Supplementary Table S1). This significance level was utilized solely for the univariate analysis and was chosen in accordance with the statistical literature (38). Variables meeting this cutoff were subsequently assessed for their combined effect on the outcome, using multiple logistic regression with a significance level of alpha = 0.05, as previously described (38).

## Results

### Effect of vibration therapy on pain severity

**Figure 2:**
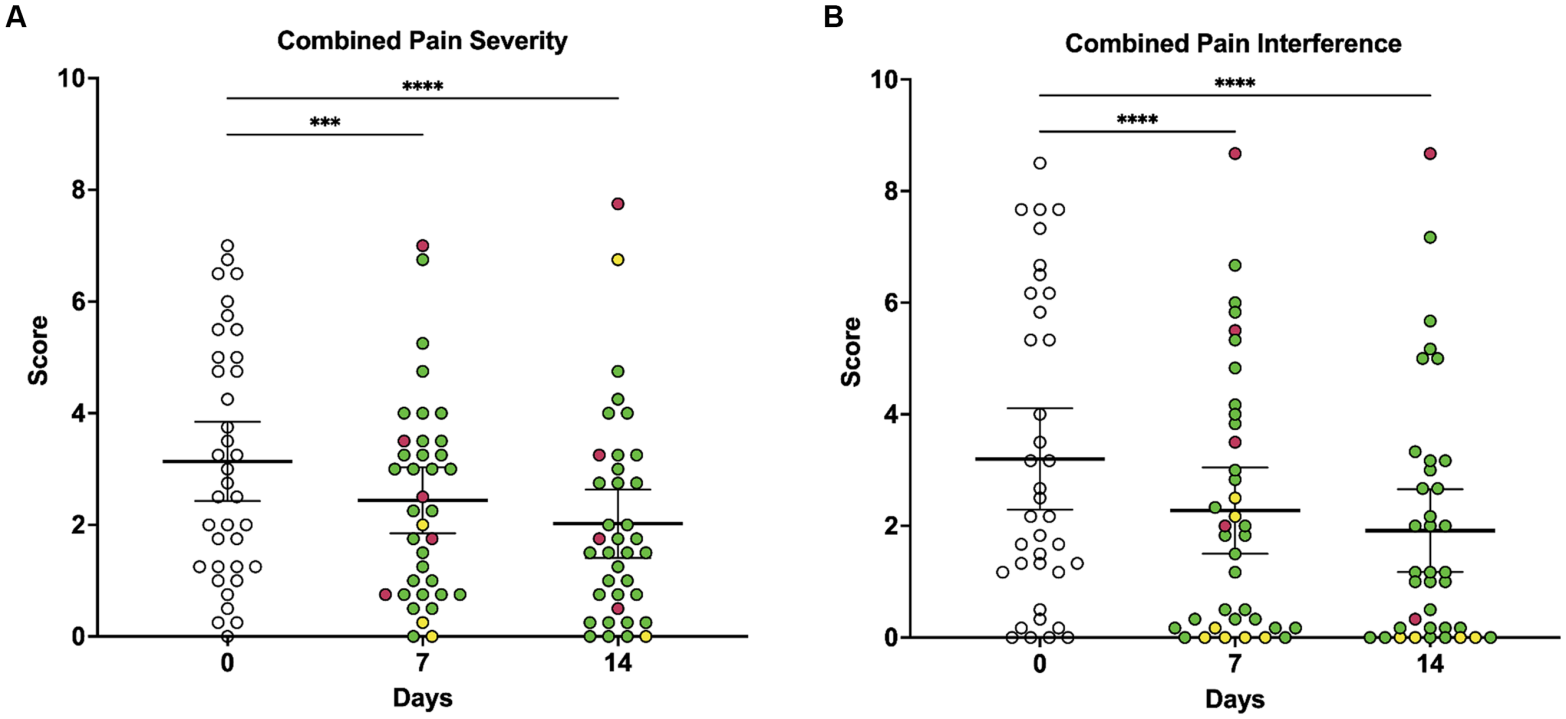
Owner-reported CBPI composite pain severity scores and composite pain interference scores at baseline, day 7, and day 14 of local vibration therapy. **(A)** Owners were asked to rate their dog’s pain level on a scale of 0 to 10 using the CBPI, and scores for the four questions on pain severity were averaged to generate the pain severity score. As assessed by repeated-measures ANOVA, average pain severity scores decreased significantly over time (*p* < 0.0001) with multiple comparisons testing demonstrating that scores were lower at 7 days (*p* = 0.0001) and 14 days (*p* < 0.0001) of LV therapy compared to baseline. **(B)** Owners were asked to rate how pain has interfered with their dog’s ability to perform different activities on a scale of 0 to 10 using the CBPI, and scores for the six questions on pain interference were averaged to generate the pain interference score. As assessed by repeated-measures ANOVA, average pain interference scores decreased significantly over time (*p* < 0.0001), and multiple comparisons testing revealed that scores were lower at 7 days (*p* < 0.0001) and 14 days (*p* < 0.0001) of vibration therapy compared to baseline. For all comparisons, the significance level is alpha = 0.05. For multiple comparisons testing, significance is indicated using asterisks: ^*^p < 0.05, ^**^p < 0.01, ^***^p < 0.001, and ^****^p < 0.0001. Each dot represents scores for individual dogs in the study over time; colors represent the change in score as compared to baseline (day 0), with green indicating a decrease in score, yellow indicating no change in score, and red indicating an increase in score. Each plot also indicates the mean and 95% confidence interval.

When owners were asked to rate their dog’s pain level on a scale of 0 to 10 using the CBPI scale, average pain severity scores were significantly lower at 7 days (*p* = 0.0001) and 14 days (*p* < 0.0001) of LV compared to baseline (Figure 2A). There was a significant overall decrease in what owners perceived to be the dog’s current pain level, as reported on the CBPI, over the course of the 14-day study (*p* < 0.0001), with multiple comparisons testing revealing significant decreases in daily pain score at days 11 (*p* = 0.0187), 12 (*p* = 0.0187), 13 (*p* = 0.0098), and 14 (*p* = 0.0041) compared to baseline (Figure 2B).

### Effect of vibration therapy on activity

**Figure 3:**
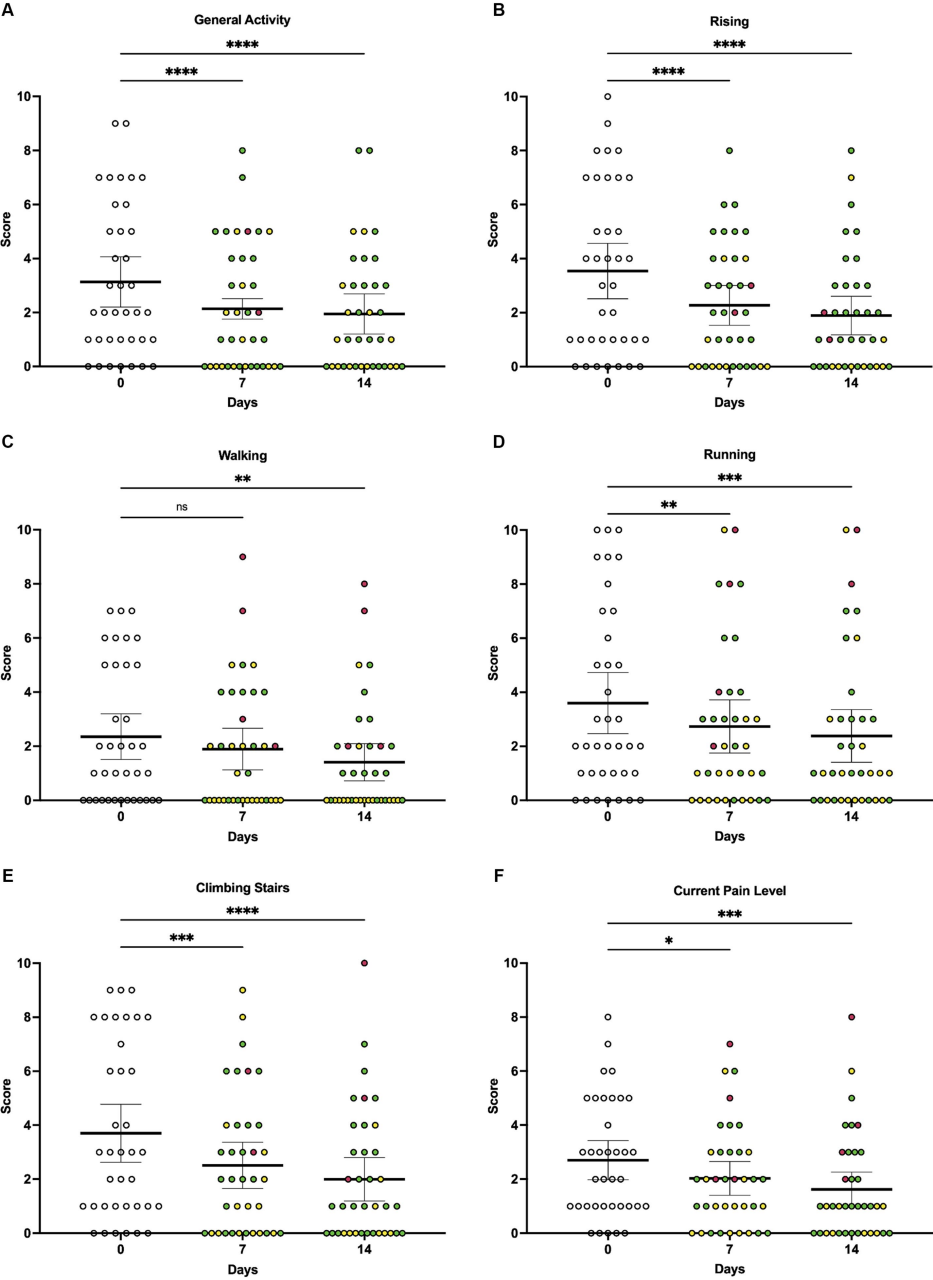
Individual owner-reported CBPI scores at baseline, day 7, and day 14 of local vibration therapy. While the CBPI is not validated for the use of individual scores to directly assess response to therapy, here we report a subset of individual scores from the CBPI to demonstrate that the significant decreases observed in composite scores were driven by decreases across multiple components of the questionnaire. For each parameter, the change in individual animals’ scores over time was assessed by repeated-measures ANOVA, with Dunnett’s multiple comparisons testing used to compare scores between baseline (day 0) and day 7 or day 14, respectively. **(A)** Average scores associated with general activity decreased significantly over time (*p* < 0.0001), with multiple comparisons testing showing significant decreases in scores at day 7 (*p* < 0.0001) and day 14 (*p* < 0.0001) compared to baseline. **(B)** Average scores associated with rising decreased significantly over time (*p* < 0.0001), with multiple comparisons testing showing significant decreases in scores at day 7 (*p* < 0.0001) and day 14 (*p* < 0.0001) compared to baseline. **(C)** Average scores associated with walking decreased significantly over time (*p* = 0.0019); while multiple comparisons testing showed no significant difference in scores at day 7 compared to baseline (*p* = 0.0813), a significant difference was observed at day 14 compared to baseline (*p* = 0.0036). **(D)** Average scores associated with running decreased significantly over time (*p* < 0.0001), with multiple comparisons testing showing significant decreases in scores at day 7 (*p* = 0.0016) and day 14 (*p* = 0.0002) compared to baseline. **(E)** Average scores associated with climbing stairs decreased significantly over time (*p* < 0.0001), with multiple comparisons testing showing significant decreases in scores at day 7 (*p* = 0.0008) and day 14 (*p* < 0.0001) compared to baseline. **(F)** Average scores associated with owner’s assessment of their dog’s current pain level decreased significantly over time (*p* = 0.0003), with multiple comparisons testing showing significant decreases in perceived current level of pain at day 7 (*p* = 0.0131) and day 14 (*p* = 0.0007) compared to baseline. For all comparisons, the significance level was set to alpha = 0.05. For multiple comparisons testing, significance is indicated using asterisks: ^*^p < 0.05, ^**^p < 0.01, ^***^p < 0.001, and ^****^p < 0.0001. Each dot represents scores for individual dogs in the study over time; colors represent the change in score as compared to baseline (day 0), with green indicating a decrease in score, yellow indicating no change in score, and red indicating an increase in score. Each plot also indicates the mean and 95% confidence interval.

When owners were asked to rate how pain has interfered with their dog’s ability to perform different activities, average pain interference scores were lower at 7 days (*p* < 0.0001) and 14 days (*p* < 0.0001) of vibration therapy compared to baseline (Figure 3A). There was a significant decrease in the perceived interference of pain with general activity at day 7 (*p* < 0.0001) and day 14 (*p* < 0.0001), rising from laying down at day 7 (*p* < 0.0001) and day 14 (*p* < 0.0001), walking at day 14 (*p* = 0.0036), climbing stairs at day 7 (*p* = 0.0008) and day 14 (*p* < 0.0001), and running at day 7 (*p* = 0.0016) and day 14 (*p* = 0.0002) compared to baseline (Figures 3B–F).

### Response to therapy

When response to therapy was defined as a reduction in both pain severity score and pain interference score compared to baseline, most dogs responded to therapy during the study period, with a response seen in 62% (23/37) of dogs to 7 days of treatment and 73% (27/37) of dogs to 14 days of treatment. Of the individuals that had responded at day 7, 91% (21/23) continued to respond at day 14. Of the dogs that did not respond at day 7, 43% (6/14) later went on to respond at day 14. 8.7% (2/23) dogs that initially responded at day 7 no longer responded at day 14.

### Predictive effects

There was no significant difference in the severity of HD score between left and right hips (*p* = 0.1024). There were no significant effects of age, sex, weight, or cumulative HD score on likelihood of response to therapy after 7 or 14 days of LV (Supplementary Table S1). Baseline pain severity score and baseline pain interference scores were considered potential predictors for response to therapy at day 7 but not at day 14. The average baseline pain severity score was higher in responders (7 days—3.61; 14 days—3.35) compared to non-responders (7 days—2.36, 14 days—2.55). Similarly, the average baseline pain interference score was higher in responders (7 days—3.76, 14 days—3.41) compared to non-responders (7 days—2.27, 14 days—2.63). However, in the multivariate analysis, the combined effect of baseline pain severity score and baseline pain interference score on response to therapy at 7 days did not reach statistical significance (*p* = 0.0522).

## Discussion

Canine HD is a prevalent degenerative orthopedic condition that causes significant and progressive lameness and discomfort in affected animals. Current therapies include medical management (39–43); surgical management, and rehabilitation modalities (44–46). Current options have limitations with regards to side effects, accessibility, and cost. Innovative and non-invasive treatment modalities to address this ailment are continually sought to enhance quality of life for our canine companions.

In recent years, vibration therapy has garnered attention as a potentially viable therapeutic option to manage orthopedic disease in humans. Only two other studies have evaluated vibration therapy for the treatment of canine orthopedic disease (32, 33). Gomes et al. (32) evaluated the effects of WBV in dogs with bilateral HD and detected significant increases in hind limb muscle mass, as assessed by multiple objective measures, as well as a reduction in pain as assessed by an owner-completed questionnaire. However, they did not appreciate significant improvements in the results of kinetic gait analysis over time. Martins et al. (33) demonstrated that patients who received one intra-articular injection of hyaluronic acid combined with WBV had better clinical outcomes, defined by quicker improvement in pain, lameness and kinetic gait analysis, in dogs with osteoarthritis due to hip dysplasia compared to dogs who received only hyaluronic acid. Both studies assessed the effects of long-term low-frequency WBV, while our study utilized short-term high-frequency LV; since our methodologies differed substantially, we cannot make direct comparisons between study results.

In particular, LV therapy is an attractive option for dogs with chronic orthopedic conditions. Home treatment reduces the financial and time cost associated with formal rehabilitation sessions while also allowing dogs to remain in a comfortable environment, reducing stress or mobility-related pain associated with transport. Our study is the first to evaluate LV therapy in dogs, with results showing a reduction in both pain severity and pain interference scores in 62% of dogs at 7 days of LV and 73% of dogs at 14 days, suggesting that LV was safe and effective in these patients. Our results also show that the response to therapy was consistent over time, with 91% of dogs that initially responded at 7 days continuing to respond at 14 days. Of the dogs that did not respond to therapy at 7 days, 43% went on to respond at 14 days, suggesting that there are benefits to completing a full 14-day trial when evaluating a dog for response to therapy.

Most patients in this study were medium, large, and giant breed dogs, correlating with the reported incidence of HD in the canine population. There were no statistically significant effects of age, sex, weight, or cumulative HD score on the likelihood of responding to therapy, suggesting that this therapy could benefit a wide range of patients regardless of age, weight, and radiographic or clinical severity of HD. Average baseline pain severity and pain interference scores were higher in dogs that responded to therapy compared to dogs that did not respond to therapy, but the difference between groups did not reach statistical significance. Nonetheless, further assessment of the effect of disease severity on response to therapy is warranted. Animals in this study likely started at different baseline pain levels, due to disease severity, concurrent ongoing management, individual pain tolerance, or comorbid conditions, all of which may influence their response to therapy. For example, if the owner’s perception was of low pain at baseline, then there would be little room for improvement in CBPI scores following LV therapy, which may lead to a conclusion that the dog did not respond to therapy. Conversely, dogs with comorbidities might be unresponsive to therapy secondary to those comorbidities, regardless of the effect of LV on orthopedic-related pain. While we did not exclude animals from the study due to the presence of other orthopedic conditions or comorbidities, we aimed to mitigate the impact of different baseline pain levels and any impacts of concurrent conditions by using each animal as its own baseline, thus assessing each animal’s response to therapy as a function of that animal’s starting score. Ultimately, the majority of dogs in the current study showed a response to therapy. There were two dogs in our study that responded to therapy at day 7 (as defined by a decrease in both pain severity and pain interference scores compared to baseline) but no longer responded at day 14. In both dogs, pain interference scores at day 14 continued to be lower than baseline, but pain severity scores no longer showed improvement. One possible explanation is that pain severity may wax and wane over time; since the pain severity score is an average score incorporating the least and the worst pain levels, it is possible that dogs with more fluctuation in their daily pain levels could have more varied pain severity scores from day to day. However, without baseline and repeat veterinary evaluations, it is not possible to speculate further about the level of pain, movement limitations and mobility as a whole.

The exact mechanisms underlying pain modulating effects of LV have yet to be fully defined, but may involve the “gate control” theory, which suggests that providing a non-noxious stimulus can reduce the transmission of pain signals along the same pathways and stop those signals from ultimately reaching the brain (47–49). Vibratory stimulation of mechanoreceptors, particularly the Pacinian corpuscles in the deep dermis, may also contribute to analgesia (48); this mode of stimulation has been posited to be responsible for 90% of gate control pain relief (49). Although there are no published studies reporting standard vibration settings using WBV or LV therapy for pain management, a systematic review of LV showed frequencies between 100–250 Hz activate the gate control mechanism more than other neurophysiological mechanisms (50). Therefore, in this study, we utilized a local vibration device with a frequency setting of 120 Hz and amplitude setting of 1 mm. However, further studies could assess a range of frequency and amplitude settings, as settings optimal for human patients may not necessarily be optimal in canine patients.

Our study relied on daily owner-conducted behavioral assessments to assess pain severity, pain interference, and response to therapy. The history of a dog’s activity level, mobility, and perceived pain is a key component in veterinary assessment of a dog’s orthopedic status, particularly when this information is provided by a person highly familiar with the animal’s behavior. However, owner assessments are subjective. In this study, we used the CBPI, which offers several benefits to traditional descriptive histories. The CBPI has been validated for use in assessment of chronic orthopedic pain in dogs, with a goal of mitigating subjectivity and promoting consistent and repeatable scoring (34). The CBPI has been used as an objective outcome measure of pain and function in dogs in numerous studies, including the efficacy of bedinvetmab for alleviation of pain associated with osteoarthritis in dogs (51, 52), the use of 117 m Tin radiocolloid as a primary treatment of canine elbow osteoarthritis (53), and the efficacy of an NSAID in dogs with osteoarthritis (36). In these studies, the CBPI was able to detect improvements in pain scores in dogs receiving treatment, indicating the sensitivity of the CBPI to changes in pain severity and pain interference; in addition, the CBPI detected larger improvements in pain scores of dogs in the treatment group compared to the control group, demonstrating the ability of this questionnaire-based system to overcome potential placebo effects associated with owner-provided responses. The CBPI also allows for frequent evaluations that occur in the animal’s typical environment. To complement the data provided by CBPI scoring, clinical examination by a veterinarian at baseline and periodically throughout the study would have strengthened this study by adding more objective data, such as visual assessment of gait, pain, and range of motion, goniometry, or force plate analysis. It should be considered that the frequency of required veterinary visits may limit enrollment; therefore, future study designs could incorporate a combination of periodic veterinary assessments as well as daily home assessments.

While the CBPI is only validated for comparisons of composite pain severity and pain interference scores, we chose to also report the changes over time in a subset of individual questions relating to pain interference, to demonstrate the depth of response to therapy. Analysis of the change in scores from individual CBPI questions over time has not been validated as a measure of pain assessment and should therefore be interpreted in the context of the overall CBPI pain severity and pain interference scores. Our results showed that not only was there a decrease in composite scores in the majority of patients after 7 and 14 days of therapy, but that strikingly, these pain severity and pain interference scores represent decreases across every parameter contributing to those scores, including more strenuous activities such as running.

A major limitation of our study design is the potential for a placebo effect (54), as owners applied the vibration therapy and performed the scoring themselves. A prior study assessing the analgesic effects of local mechanical vibration in humans with knee osteoarthritis included an active treatment group that received treatment in the form of a knee brace with integrated heat, vibration, and a foot pedal that delivered continuous passive motion (55). The control group received a sham, which consisted of a non-vibrating, non-heating knee brace with no foot pedal (55). We considered using a similar sham treatment, in which a nonfunctional device (e.g., a device without batteries) would be applied by owners daily. However, we elected not to utilize this format because the handheld vibration device used in this study emits physical vibrations that the owner can feel while applying the therapy, therefore we found it likely that owners would discern whether they were assigned to the treatment or placebo group. While it would theoretically be possible to provide the device to owners without stating the mode of therapy it provides, therefore allowing some owners to consider that the sham device might still be providing a therapy that they are not able to see or feel, we felt it would be difficult to ensure owners would remain blinded to the therapy since the device in our study is available on the market. This would be a confounding factor in interpreting data produced from such a sham group. In addition, we did not enroll dogs without hip dysplasia in the study. Given these limitations, further studies are necessary to assess the true benefit of LV for dogs with HD, and should be randomized, blinded, and controlled. This could be achieved by having another party apply (or not apply) the device, and having the owner score the dog using the CBPI without knowing whether the dog received treatment. One method of implementing such a study design would be to enroll dogs with two owners in the household, one of which would apply therapy and the other which would complete the CBPI; however, this would limit enrollment and would also require compliance of the owners not to share information with each other. Another method of implementing this blinded method would be to have a third party apply therapy at a central location, such as a veterinary clinic or rehabilitation center, allowing owners to then complete the CBPI while blinded to the treatment group. However, this would require owners to travel, potentially reducing the feasible frequency of treatment, limiting enrollment to owners that can incur the time and financial cost, and restricting participants geographically. In addition, certain dogs may be more stressed or excitable in an external setting and may not be as willing to accept vibration therapy.

Another major limitation of working with client-owned animals is the inability to verify owner compliance throughout the study period. As the vibration device was applied by the owner and use was not supervised by the organizers of the study, variations in owner diligence, understanding, and adherence to instructions may have influenced the consistency in application throughout the study period, and overall efficacy of therapy. To reduce the risk of non-compliance, owner training was provided in the form of video demonstrations and written instructions, and reminders to apply therapy were delivered via daily emails. Future studies could also refine training mechanisms by including hands-on tutorials or follow-ups to ensure consistent device application. Similarly, owners were instructed to continue their dog’s normal exercise regimen and medications throughout the study period, but we were not able to monitor compliance beyond owner affirmation. Therefore, it is possible that the response to therapy in some animals was affected by increases in activity level, acquisition of new injuries, or use/disuse of medication or other therapeutics. It is therefore difficult to identify the cause for failure to respond to therapy in this population. While sudden increases in activity level could potentially affect the response to therapy, we do not anticipate that decreases in activity level could produce the opposite result (i.e., a false decrease in pain score). In the authors’ clinical experience, rest alone is unlikely to improve pain associated with HD given the pathogenesis of the disease. The potential effects of inter-user variation in individual application of vibration therapy or CBPI application are also mitigated by the comparison of post-treatment scores to each dog’s individual baseline scores.

Despite limitations, this study contributes important information to the literature as the first study to investigate the potential for LV use in dogs with orthopedic disease. We found that daily LV was associated with decreased pain severity and pain interference, providing initial support for further investigation of the use of LV therapy in dogs with bilateral HD. Canine HD remains a significant concern in the veterinary community, necessitating innovative therapeutic interventions. Further comprehensive studies, especially those involving larger sample sizes, control/sham groups, and blinding, are important to fully assess the value of LV in the management of canine orthopedic disease. The assessment of longer courses of treatment and the application of LV for different orthopedic conditions should also be considered. In addition, the incorporation of multiple measures, such as periodic veterinary orthopedic examinations or force plate gait analysis, may complement the results of owner-conducted behavioral assessments. LV via handheld devices presents a promising future avenue for alleviating discomfort and enhancing mobility in canine orthopedic diseases, especially when combined with a multimodal management approach.

## Data Availability

The original contributions presented in the study are included in the article/Supplementary material, further inquiries can be directed to the corresponding author.
