# Corrigendum: Client-based evaluation of the effects of localized vibration therapy on pain and mobility scores in dogs with radiographic bilateral hip dysplasia

**DOI:** 10.3389/fvets.2024.1503011

**Published:** 2024-10-22

**Authors:** Kristal F. Turner, Sherman O. Canapp, Debra A. Canapp, Angela M. Sutton, Allyson Canapp, Isabel A. Jimenez, Joyce Gerardi

**Affiliations:** ^1^Canapp Sports Medicine, Oakland, MD, United States; ^2^mOcean mobility + wellness for animals, Jacksonville Beach, FL, United States; ^3^Johns Hopkins University School of Medicine, Baltimore, MD, United States; ^4^Synergy Integrative Speciality Veterinary Clinic, New Bern, NC, United States

**Keywords:** vibration, vibration therapy, localized vibration therapy, pain management, hip dysplasia, vibration treatment, canine (dog), rehabilitation

In the published article, there was an error in the Funding statement.

The funding statement was displayed as “The author(s) declare that no financial support was received for the research, authorship, and/or publication of this article.”

The correct Funding statement appears below.

