# Supplementary material for: Client-based evaluation of the effects of localized vibration therapy on pain and mobility scores in dogs with radiographic bilateral hip dysplasia
Source: Front Vet Sci. 2024 Aug 21;11:1424373. doi: 10.3389/fvets.2024.1424373 (PMC11373568; doi:10.3389/fvets.2024.1424373)
Supplement: Supplementary file 1 [file Table_1.docx]

**Supplemental Table 1: Assessment of potential predictor variables for response to therapy, as assessed by univariate logistic regression**

| Variable | Response to therapy at 7 days | Response to therapy at 14 days |
| --- | --- | --- |
| Age (years) | p=0.4810 | p=0.1609 |
| Sex (Male vs. Female) | p=0.5837 | p=0.8374 |
| Weight (kg) | p=0.7187 | p=0.9591 |
| Cumulative HD score | p=0.3316 | p=0.6941 |
| Baseline pain severity score | p=0.0522 | p=0.1823 |
| Baseline pain interference score | p=0.0522 | p=0.1157 |
